# Zoonotic anxieties: The cultural politics of Nepal's quest for pandemic preparedness

**DOI:** 10.1111/maq.70025

**Published:** 2025-10-22

**Authors:** Max D. López Toledano, Hari Basnet, Anna Durrance‐Bagale, Natasha Howard

**Affiliations:** ^1^ Saw Swee Hock School of Public Health National University of Singapore and National University Health System Singapore Singapore; ^2^ Natural History Museum Denmark University of Copenhagen Copenhagen Denmark; ^3^ London School of Hygiene & Tropical Medicine, Department of Global Health & Development London UK

**Keywords:** disease surveillance, health security, Nepal, one health, pandemic preparedness

## Abstract

Based on fieldwork conducted in Nepal (2022–2024) and by paying attention to how local and transnational notions of epidemiological risk are deployed, this ethnography introduces the concept of “zoonotic anxieties” to make sense of the multi‐species relational ethos that contemporary global health regimes propose. Namely, at the intersection of “health security,” “pandemic preparedness,” and “One Health,” multispecies interactions are becoming subject to a resignification where cultural, racial, and geopolitical anxieties are deposited, expressed as fears over the potentially apocalyptic emergence of zoonotic disease. Nepal is produced through this lens as an epidemiologically risky terrain, receiving funds from international agencies to enhance its “disease surveillance” capacity with the underlying hope that, by identifying infectious diseases early, a foretold global disaster can be contained. These investments, however, have had little impact, producing conflicts between the country's claim to medical modernity and the shortcomings of its disease surveillance ambitions.

## Abstract (Nepali)

सन् २०२२ देखि २०२४ सम्म नेपालमा गरिएको स्थलगत अध्ययन र स्थानीय तथा अन्तर्राष्ट्रिय स्तरमा महामारीसम्बन्धी जोखिमको अवधारणालाई कसरी प्रयोग गरिन्छ भन्ने विश्लेषणका आधारमा, यस अध्ययनले ‘जूनोटिक चिन्ता’ भन्ने अवधारणालाई अघि सारेको छ। यसले समकालीन विश्व स्वास्थ्य प्रणालीहरूले प्रस्ताव गर्ने बहु‐प्रजातीय सम्बन्धबारेको सोचलाई बुझ्न सहयोग गर्छ। विशेषगरी ‘स्वास्थ्य सुरक्षा’, ‘महामारी तयारी’ र ‘एक स्वास्थ्य’ को अन्तरसम्बन्धमा बहु‐प्रजाति अन्तरक्रियाहरूलाई नयाँ अर्थ दिइएको छ, जहाँ सांस्कृतिक, जातीय र भू‐राजनीतिक चिन्ताहरू संचित भएर जूनोटिक रोगको सम्भावित विनाशकारी उदयप्रतिको भयका रूपमा व्यक्त गरिन्छ । यस दृष्टिकोणले नेपाललाई महामारीजन्य जोखिमयुक्त भूभागका रूपमा चित्रित गरेको छ, यसले अन्तर्राष्ट्रिय निकायहरूबाट रोग निगरानी क्षमताको विकासका लागि सहयोग पाउँछ। यसको आधारभूत अपेक्षा भनेको संक्रामक रोगहरूलाई चाँडै पहिचान गरेर सम्भावित विश्वव्यापी प्रकोपलाई नियन्त्रण गर्नु हो। यद्यपि, यसको खासै प्रभाव परेको छैन, जसले गर्दा देशको चिकित्सीय आधुनिकताको दाबी र यसको रोग निगरानी महत्वाकांक्षाका कमजोरीहरू बीच द्वन्द्व सिर्जना गरेको छ।

What does it take to be brave and accept the risk of infection or pain, not as a way to succumb to fatalism about the finitude of antibiotics in the face of virulence but as a way to live out an ethics of decolonization, one that knows all too well the fragility and diversity of all inhabitants of this planet?

Juno Parreñas (2018)

## INTRODUCTION

Despite being in no way involved with the military, Dr. T surprised us by describing himself as a “soldier” in Nepal's “fight” against emerging infectious diseases. We met him in the quiet backroom of a restaurant‐and‐tea‐house near the hospital where he works in Kathmandu—here, he presented to us an extensive overview of Nepal's infectious disease landscape, encompassing the emergence and re‐emergence of H5N1, zika, brucellosis, leptospirosis, COVID‐19, Nipah virus, a potentially new strain of dengue he claimed to have identified a few weeks earlier, or even polio, which saw a vaccine‐derived resurgence in Nepal mid‐2024. Altogether, he framed these as “time bombs” in need of being diffused, emphasizing how delicate he perceives the matter at hand to be.

This “time bomb” was not entirely a metaphor. In 2020, during the COVID‐19 pandemic, Nepal tasked its army with the procurement of medical supplies from China and curfew enforcement, stirring debates over the blurred boundaries between the military and public health (Angbo, 2021; Gautam, 2020; The Record Nepal, 2020; Giri & Ghimire, 2020). After all, following 10 years of armed conflict detonated by the Maoist Insurgency, “the legacy of political revolt and violence by political parties, groups and communities, and political suppression by the state has remained a crucial problem” (Sharma Wagle, 2020). How exactly did the boundary between public health and “security” sectors erode in a country still scarred by a civil war that ended less than 20 years ago, and with what implications?

In retrospect, our surprise may not have been fully warranted. It should come as no surprise to medical anthropologists that health is framed as a military affair, considering Emily Martin's (1990) work on militaristic sociocultural attitudes toward disease or the prolific literature following the emergence of “health security” over the past three decades (e.g., Elbe, 2018; Lakoff, 2017; McLean, 2024). It is the latter which Dr. T's words appear symptomatic of, broadly referring to an array of policy‐ and discourse‐based processes that locate various aspects of public health within an ever more pervasive global “security” framework, holding as both premise and outcome that health is a significant domain of national and human security. The motif of “pandemic preparedness” has risen at the center of this biosecurity landscape, a horizon that is attuned to a “molecular vision of life” (Elbe, 2018) and in which microscopic agents have the power to direct political agendas, galvanize emergent industries, and instill new biopolitical disciplines.

Importantly, enacting “preparedness” is a transnational quest, sustained by a myriad of global investments anticipating what the World Health Organization (WHO) speculatively terms “Disease X” (WHO, 2018). Preparedness translates as a chain of geopolitical efforts to prevent, identify, and contain hypothetical outbreaks that are imagined with the potential to destabilize countries, regions, or even the whole world. Local and global public health priorities have shifted (Frankfurter, 2019), resulting in new streams of funding that constitute, to a great degree, the meaning that “health security” and “pandemic preparedness” acquire in the Nepali context. In 2023, for instance, Nepal was announced as a recipient of up to US$25 million across the World Bank's “Pandemic Fund” and United States Agency for International Development's (USAID's) “Global Health Security Programme” (USAID, 2023; WHO, 2024), joining over 50 other countries within the scope of the latter. The broadly defined ideal of “disease surveillance”—generally referring to a systematic search and documentation of diseases—has been the main target of these interventions, following the country's poor score in this domain as per the WHO‐led “Joint External Evaluation” in 2022.

The underlying suspicion is that, without assertive investment in disease surveillance, new and existing pathogens will emerge and remain undetected until they spread beyond Nepal's national borders. In other words, until it is “too late.” Global “peacetime” would be threatened, as life outside of health emergencies is increasingly framed in many—privileged—parts of the world. Moreover, this view has been furthered by the advent of “One Health” as a dominant global health perspective, given its premise that “infections are best addressed through a unified approach that brings human and nonhuman animal health together” and its consequent emphasis on the risks posed by “zoonotic” diseases (Porter, 2019, 14). Seemingly, that approximately 75% of all pathogens affecting humans in the past three decades have originated from other species has become a primary tagline in the latest chapter of international disease governance. Through this way of seeing, every interaction between humans and other animals in Nepal is a “security” risk, not just for the bodies involved in the exchange, but for the entire world.

Risk management, it would follow, ought to be the tone of life in a country where 70% of the population is involved in agricultural activities and in frequent contact with livestock of many kinds (Mishra, 2023). Expectedly, this perspective was a common denominator among the 20 health and livestock professionals in Nepal we interviewed, a majority of whom are highly concerned by the country's perceived vulnerability to hypothetical disease outbreaks. Yet, beyond those immediately working on the issue, most people in Nepal do not seem to share this concern, generally dismissive of international health agencies’ perceptions of the country as an epidemiologically risky terrain. This was a consistent attitude across the 37 interviews and 5 focus group discussions we conducted with community members in Kathmandu, Pokhara, Chitwan, Mustang, Gulmi, and Bhaktapur between 2022 and 2024,^1^ best exemplified by an interviewee from Pokhara. When discussing state‐led awareness campaigns on the risks posed by zoonotic diseases, she said: “I don't care for it. Our animals and we haven't been sick in a long time (…) The rangers from the forest and sometimes others come and give us some information. But we don't give that much attention.”

Clearly, there are disparities in perceptions of risk at different national levels and in the way global health agencies make sense of zoonotic diseases, a disparity that has become central to what this ethnography sets out to understand. How are the risks posed by “emerging zoonotic diseases” conceptualized at local, national, and transnational levels, and what are the by‐products of potentially divergent notions of risk? What transformations might be sought—and possibly achieved—by interpreting the potential emergence of disease as a “security” issue? Do they map onto the warnings issued by critics (McCoy et al., 2023) who see “neo‐colonial” interests in this deployment of the vocabulary of health? What does Nepal's participation in the global tapestry of pandemic preparedness represent, and how does this interact with the country's broader culture of care?

### ZOONOTIC ANXIETIES

As a heuristic to answer these questions, we propose the concept of “zoonotic anxieties.” By this, we simply refer to an anxious state of response that the potential emergence of zoonotic disease mobilizes among local and global public health actors. What the concept may also highlight are the broader social, political, and cultural insecurities that are stirred by the threats posed by real and imagined zoonotic diseases. Such anxieties are typically located in the perspectives of public health experts whose professional and epistemic attachments align them with shifting global health regimes, but also go far beyond. Namely, we suggest that zoonotic anxieties prompt transformations to our multispecies relational ethos and may be generative of new horizons of the human, taking as premise that “human nature is an interspecies relationship” (Tsing, 2012). What changes to the notion of “the human” are agitated when we lean into the idea that deadly pathogens are our political enemies and we expect them to emerge from any animal, any place, and any time?

Indeed, anthropologists of “preparedness” have shown how such anticipatory narratives are underpinned by feelings of insecurity, precariousness, unpredictability, and vulnerability against “bio‐terrorist” threats (Lakoff, 2017; Masco, 2014; Sharp & Chen, 2014; Wolf, 2017). Historians of science and medical anthropologists have, likewise, demonstrated how medical notions of “surveillance” transcend the scope of “health” itself, showing state intrusion on everyday life, transnational mobility, and the making of race and marginality as some of the broader forces at play (Abi‐Rached, 2021; Anderson, 2006; French, 2009; Lyttleton, 2018; Nading, 2014). Anthropologists of One Health, on the other hand, have brought a much‐needed multispecies perspective to these issues, though have largely focused on governance of diseases already in circulation (e.g., Nadal, 2020; Porter, 2019) rather than on the speculative dynamics entailed by the potential for re‐/emergence of new and old zoonotic diseases.

What has been minimally included, and what we thus seek to account for, is an angle that observes not only how human relations are transformed and re‐imagined by speculative projections of our epidemiological futures, but also how our relationships with other species are factored in. Importantly, we build directly on Fearnley's (2020, 87) notion of “vital uncertainties”, through which he suggests that multi‐species entanglements express an uncertain mode of life as potential disease stalks a “future that might come to be: gain or loss, wealth or ruin.” Our contention is that it is not just “uncertainty” but a relentless affect of “anxiety” at the interface of human/non‐human interactions which thrives in the multi‐species relational ethos of contemporary global health. “Anxiety,” after all, pertains not just to an uncomfortable uncertainty but to a “defense system [that] detects anticipated threats to a future goal and (…) calls the cognitive system into action to decide [how to] best to get rid of the threat” (Ojala et al., 2021, 38).

We thus use the term zoonotic anxieties as a proxy for the affective politics of pandemic preparedness, within which the boundaries of “the human” are being redrawn. This, of course, unfolds within the existent imperial cartographies of global health, wherein risk is spatialized and assumed to exist within certain geographies, bodies, and ecologies (Da Silva et al., 2023; Fearnley, 2020; Hinchcliffe & Ward, 2014; Lyttleton, 2018; Ong, 2008). Or, as White (2023) calls it, within a long durée of “epidemic orientalism.” The result is a tiered and hierarchical system of “humanity,” wherein the surveillance of disease in certain ecologies translates to an interrogation of whole populations’ claim to “the human,” at risk of being reduced to what Ong (2008) terms “zoonotic zones”: spaces where disease is allowed to circulate but where it ought to be contained, commonly by restricting the mobility of living organisms—human or not—away from imperial metropoles.

The search for “the next pandemic” thus appears to be a self‐reinforcing political project grounded in (primarily Western) “bio‐insecurities” that only ever become more specific, creating a self‐referential need to invest in the very same worldview, technologies, and relationships that these insecurities stem from. Masco (2014, 23) notes: “Perfect [preparedness] (…) is an imaginative horizon projected onto a deep future and crafted by experts rehearsing catastrophic endings with ever‐greater precision.” To seek this precision is to embark on a never‐ending quest, an exhaustive pursuit toward the elimination of risk where the only guaranteed outcomes are feelings of “insecurity” and “vulnerability.” The global health regimes at hand, in that sense, are cause and cure of their own malaise: self‐fulfilling prophecies that in the quest to secure, “create” the very same epidemiological, geopolitical, and racial insecurities they claim to solve, all packaged and sold as anxieties over the potential spread of real and imagined zoonotic diseases.

## THE PROPHETIC EPISTEME OF HEALTH SECURITY

In April 2019, under mysterious circumstances, 50 to 60 crows were found dead near the British Embassy in Kathmandu. In fact, reports suggest that over 300 crows died during that period (Kathmandu Post, 2019). Viral samples were sent to Australia and Japan for post‐mortem laboratory assessments, and Dr. T warned in his column: H5N1, commonly known as Bird Flu, could be returning after a 2‐year hiatus, as no human cases had been reported anywhere in the world since 2017. This was the most compelling—if not the only—explanation for the mysterious series of bird fatalities, he argued. Shortly after, his prophecy was fulfilled. Laboratory tests in Japan attributed the crows’ death to H5N1 and, by May 2, 2019, responsibility was attributed to the virus for a 21‐year‐old truck driver residing in Bhaktapur who died on March 29, more than 1 month prior (Kathmandu Post, 2019). The government deployed an investigative team, although Dr. T believed this had been too late. Luckily, the virus did not spread further, and the world was spared what could have become a pandemic approximately one year before COVID‐19 emerged and interrupted “normal” life in most parts of the world.

However, in the way Dr. T spoke, there was an intriguing tone of inevitability. As he recounted this story, he clearly prided himself on his predictive powers, but above all used this anecdote to demonstrate his concern and provide evidence for what felt to him like a certain fact: that infectious diseases as devastating as COVID—or perhaps even more—await our common future. Other health professionals echoed this vision, and this way of making sense of the future is far from exclusive to Nepal. When analyzing data, holding discussions, and working on this project outside of the country, it was impossible not to gather a seemingly endless list of hypotheses on one of the most provocative questions for global health academics and practitioners: what will “the next pandemic” be and where will it come from? Many scholars will point at global food production systems (Porter, 2019), reliant on high‐density animal farming, to suggest that Bird Flu or a disease transmitted by pigs are most likely to be the “next Covid”; dengue's “pandemic potential” is an accelerating concern (Nading, 2014); bats are increasingly discussed as flying pandemic threats (Reuters, 2025); the transmission potential of mpox—declared “public health emergency of international concern” in 2022 and 2024—remains largely unclear (Reardon, 2024); and many health professionals in Nepal are very concerned with the potential of the Nipah virus, found in recent years among bats near the open border with India and Bangladesh and described as potentially catastrophic if it were to spread at a larger scale.

These are all samples of what we refer to as the “prophetic episteme” of health security, wherein imaginative constructions of foretold epidemiological disaster are scientifically and politically mobilized to prime society in an anticipatory manner. An “episteme,” Foucault (1966) argued, provides the grounds for scientific knowledge in frequently hidden configurations, acting as the “unconscious of science.” Prophecies, on the other hand, imbue some people—public health experts—with the power to “see what others cannot see” and “prompt people to place their lives into the[ir] hands” while grounded in speculative ways of knowing (Caduff, 2014). Thus, while narratives anticipating “the next pandemic” are typically coated in the legitimacy of scientific discourse, social scientists have insisted that projects that anticipate and prepare for disaster largely depend on speculation, uncertainty, prophecy, imagination, or even faith to forecast worst‐case scenarios of the future that *may or may not* come true (Caduff, 2014; Chen & Sharp, 2014; Cooper, 2006; Lakoff, 2017; Lyttleton, 2018; Masco, 2014; Wolf, 2017).

Pandemic “preparedness,” then, untethers epidemiological response plans from any specific disease, mobilizing vast amounts of funding, political will, and coordination efforts in the quest to end the next pandemic “before it even begins” and “govern a future that cannot be cut down to calculable forms” (Wolf, 2017). So, if uncertainty over when, where, how, or even *whether* the “next Pandemic” will happen seems to be such a central aspect of preparedness, how had Dr. T—in unison with most health professionals we spoke with—come to attribute such an inevitable feel to it? More broadly, if modern medicine purports to have become “evidence‐based,” why do health systems seem to be increasingly guided by political visions that are largely rooted in the “unknown”? What are the implications of defining and governing the future in relation to such uncertain visions, allowing them to determine our healthcare priorities and cultural, political, and affective forms?

While the idea of “pandemic prophecy” has circulated for over a decade (Caduff, 2014), we understand it as merely the epidemiological arm of a larger body of apocalyptic prophecies that increasingly dominate and foster insecurity in the Western mind (Bounds, 2020; Chen & Sharp, 2014). Gone are the days of imagining the future as “reliable,” “trustworthy,” and “better than the present” that characterized the modern era (Berardi, 2011). The political limits of “hope” and “optimism” have been exhausted (Berlant, 2011). Pessimism and a “refusal of the future” (Edelman, 2004) have emerged as new affective and political directions, amounting at large to what Mbembe (2024, 2) refers to as “collapsology”: the narratives and studies that stem from the standpoint where we “no longer believe in the future,” where “we no longer expect anything, except the end itself.” Cultural products in Western mass media offer recurrent expressions of this, producing in recent decades a wealth of imagery depicting apocalyptic scenarios of any imaginable kind. In the case of pandemics, this is represented by the exorbitant rise of the “zombie” as “epidemiological avatar” in science fiction, expressing growing feelings of insecurity that are embodied by “sick” and “enemy” bodies who are dangerously infectious, difficult to contain, and have the power to uproot the social order across the world (Verran & Reyes, 2018).

**FIGURE 1:**
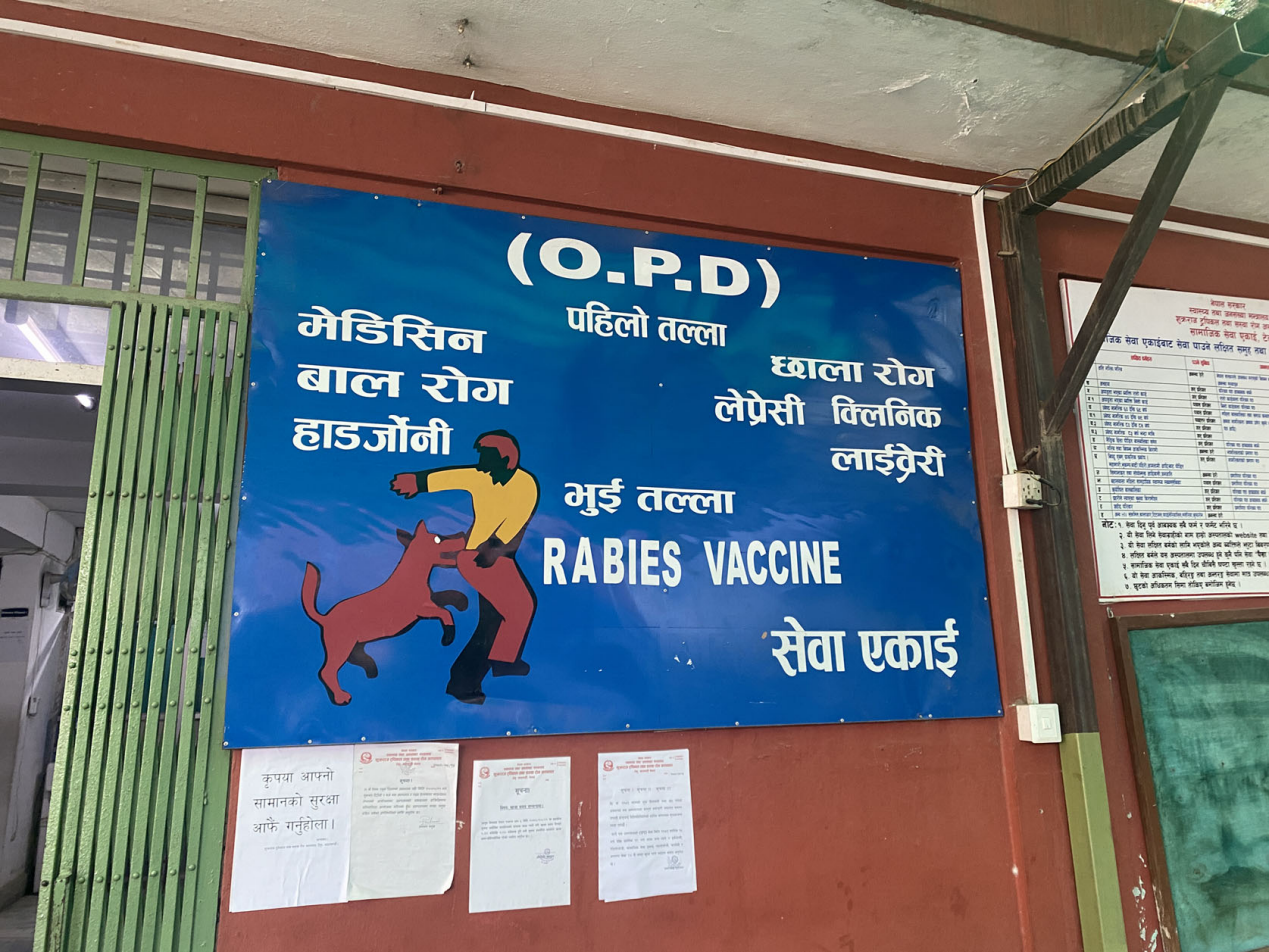
Rabies vaccination site at a hospital in Kathmandu. Photo by author.

Nevertheless, Western apocalypse—or “Settler Apocalypse,” as Kim TallBear (2023) calls it—is not everyone's prophecy. In our interviews with community members in Nepal, many of whom are “at risk” due to frequent contact with animals that may act as disease vectors, we found hardly any trace of the concerns that have become so central to health security's view of the world. Across Nepal, zoonotic diseases are barely a concern for anyone not working directly in the health or livestock sectors, with rabies being the main exception (Figure 1). If anything, people care about rats and their immediate impacts, for these eat their grains, damage their clothes, and excrete waste in their homes. However, rarely will they be pre‐emptively concerned with the potential diseases these animals carry and may expose them to (e.g., leptospirosis). Most people simply cannot afford to be concerned—having little control over their means of livelihood and the socioecological context in which they live—and are instead reluctant to embrace perpetual insecurities that are not their own and that they most likely can never be fully protected against.

Accordingly, skepticism and doubt were common responses to the conceptual risks of zoonotic diseases throughout our interviews. Most people have limited awareness and do little to mitigate risks they perceive as largely speculative, as a participant from Gulmi exemplifies: “Rats have been around us since a long time, we also see them in the field, but we have never fallen sick. Do they really transmit disease?” Everyday proximity and coexistence with other animals are the norm in most of Nepal, and animals will be assumed to be healthy if they look and feel that way: “I have reared them and they are healthy and I don't think they would transmit disease,” explained a different interviewee from Gulmi.

Cautionary measures may not be the most popular even during known outbreaks. For instance, while discussing the poultry culling that accompanied past H5N1 outbreaks, a participant from Pokhara confessed: “Once, during Bird Flu, people started selling their chickens at cheaper prices instead of killing them,” noting that the government does not always compensate people for potential monetary losses when they are expected to cull their animals. COVID‐19 did make people “more cautious,” as per a 51‐year‐old woman from Pokhara, but fears of pandemic catastrophe do not seem to have reached most parts of the country, for vaccine hesitancy was reportedly common among health‐workers during the COVID‐19 pandemic. A physician working in a public hospital explained the hesitancy observed among colleagues: “Government said ‘go and get a vaccine’, but never sufficiently explained why you need a vaccine, that is the problem. Before, nobody went to get vaccinated: ‘Why should I have to get a vaccine?’, people asked every time.”

The zoonotic anxieties that cast Nepal as a potential source of the pandemic apocalypse, it seems, are not shared by everyone in the country. And similarly, not all pessimism is the same. As Indigenous and post‐colonial scholars have noted (Dalley, 2016; Parreñas, 2018; TallBear, 2023), contemporary visions of the apocalypse and fears of “extinction” largely respond to a colonial view of the world whose concerns are rooted in the demise of Empire, all while imbricated in the mixture of the “prophetic with the profitable” that has become endemic to “disaster capitalism” (Caduff, 2014). The apocalypse is an industry, and its business model lies in relocating “hope” away from prevention towards containment instead, an ethos described as the ideological pillar of health security (McCoy et al., 2023). Thus, understanding pandemic prophecy in Nepal as an import product, we ask: How does pandemic prophecy express itself in Nepal, and, if it is a product of Western sociopolitical pessimism, what is at stake in making its fears our own?

### DISEASE SURVEILLANCE IN NEPAL, OR LACK THEREOF

As medical anthropologists have already noted (Fearnley, 2020; Frankfurter, 2019; Nading, 2014), disease surveillance occupies a central role in any aspiration to know and respond to the next pandemic. Nevertheless, despite the influx of resources to bolster Nepal's surveillance capacity, this proved to be a rather elusive figure throughout our fieldwork. Time after time, we encountered the claim that “disease surveillance is minimal” or occasionally even the extreme “there is no disease surveillance in Nepal.” By the end of data collection, we gathered a long list of explanations as to why disease surveillance in Nepal is deficient, including: poor database management in clinics, constrained laboratory resources to study viral samples, restricted access to the internet or technical equipment in health facilities, incompatible surveillance reports and/or unwillingness of private health providers to share their data, or, most frequently, simply as necessary by‐product of the constraints on the health system as a whole.

From early on, it became clear that making sense of Nepal's quest for preparedness must be paired with an understanding that the country's capacity for care is limited and highly unequal. Approximately 80% of Nepal's population lives in rural settings (Adhikari, 2020), of which approximately 41% has no access to health posts and 80% has no access to public hospitals within 30 min of their home using public transport (Ashworth et al., 2019). Availability of health services is strongly demarcated by an urban‐rural divide that makes access to state‐provided healthcare significantly inconvenient, unaffordable, or impossible for most, given the need to travel to cities for quality care. The uneven mountainous terrain that shapes the country's topography, likewise, imposes major limits on mobility outside and between urban settings. For instance, if someone living outside of the Kathmandu Valley must access medical resources only available in the capital, this could potentially require long hours of transport, queueing, and staying overnight—or potentially multiple nights—before they can make their way home. Most of the population remains outside of the scope of the health system, accounting for claims throughout our interviews, such as “the hospital is too far” to justify not visiting health facilities, or to people simply not knowing where the nearest facility is.

As a result, much of Nepal's population relies on Ayurvedic traditions, traditional healers, community‐based services (including the female community health volunteer network), and home remedies before considering the state's system for health concerns. A participant from Mustang explained that “most people try home remedies to cure certain diseases (…), then, if it does not work, we take them to the health post.” Another participant from Chitwan echoed: “We have visited the traditional healers in the past when my children were small because it was believed they can heal them. In case that did not work, we would take them to the hospital.” Participants from Bhaktapur and Pokhara also alluded to this, suggesting that, for some people, the default health response is to seek help from *jhakris* or “shamans” when bitten by animals, including dogs. Managing the health of other species is much the same, as a participant suggested:

“People ask us to take [our animals] to the veterinarian, but to get there it takes three to four days. Until then, the buffaloes may already be healthy or have died due to the sickness. There are no proper services here. We give them garlic clove, mustard seed, and other weeds.”

Therefore, for many people in Nepal's health sector, the idea of surveillance of zoonotic diseases feels like a distant dream. Quite simply, as a veterinarian claimed, when it comes to zoonoses surveillance, “no one knows what's going on.” This was accompanied by “insecurity,” as tone and feeling, in the way our participants described the country's ability to respond should an outbreak occur. Among healthcare and livestock professionals, the absence of a robust disease surveillance system produces uncertainty and speculation, nurturing multiple cultural anxieties that undergird their perceptions of Nepal as an epidemiologically “risky” terrain. These anxieties, we argue, are endemic to the preparedness regime, and result in certain cultural groups being cast as epidemiological threats when its lens is adopted and internalized by domestic actors.

Across our interviews, health professionals repeatedly insisted on the need to increase awareness of zoonotic diseases among rural populations, primarily attributing the country's shortcomings to widespread “ignorance.” This emphasis constructed the challenges of pandemic preparedness in Nepal as knowledge‐ and culture‐based issues, wherein educating and reforming rural and “traditional” classes would most effectively produce the desired outcomes. Consequently, we encountered latent conflicts between the way cultural traditions are perceived and the modern demands of health security, alongside a broader series of antagonistic relationships. According to this group of participants, cultural practices and rituals interfere with proper risk management, sanitation is incompatible with rural life‐ways, and, overall, medical modernity requires the effacement of tradition. For example, a veterinarian argued that, in case of a hypothetical “Foot and Mouth Disease” outbreak, Nepal would face cultural challenges:

“Most people in Nepal are Hindu, so they don't have a policy to slaughter cows. That's a big problem. Even brucellosis. Because we have brucellosis in goats, even cattle. So, as we don't have the slaughtering policy for the [cows], it's very difficult to control the disease.”

The same participant referred to other cultural traditions as zoonotic risks:

“In Mustang, they have a festival, they have a tradition… They don't slaughter the yak, they cut the neck and drink the blood, and they believe that it makes them healthier (…) Rats have many diseases. We have reports of leptospirosis in animals and in humans. So that might be a burden, but people have a culture. They have a tradition that after they harvest the [rice] paddy, they keep on digging and digging to get the rats. They make the rat like a barbeque.”

Similarly, a district‐level health official explained:

“90% of people don't use latrine. They go in the field for open defecation, and that's why we faced a cholera outbreak last year. More than 2,000 people were affected, seven people lost their lives because of the cholera outbreak. In the 21st century. And what I was talking about with the political leaders here is, John Snow studied cholera in the UK around 200 years back, and we are doing it now. It means we are 200 years back compared to the UK.”

Zoonotic disease surveillance was an evocative topic for this group of participants, revealing deeply rooted cultural anxieties. In fact, we may even think of these as part of the “indirect repercussions the steps taken to pursue development have on everyday life,” to which anthropologist Stacy Pigg (1993, 46) has long directed our attention when studying Nepal. After all, these depictions of rural classes locate epidemiological risk within cultural patterns and rely on discursive separations based on an “us” versus “them” model that is driven by explicit affinities to a sanitary kind of Western modernity. Through the production of epidemiological “Others” in rural groups, participants constructed and presented an image of the modern Nepali subject—urban, wealthy, healthy, “clean,” and with a global outlook—projecting a vision of a “sanitised” and “secure” Nepal, cleansed of traditional markers that its “backward” and “uncivilised” rural communities allegedly uphold. As Said (1979) notes in *Orientalism*, such representations have been central to the discursive construction of modernity and, more specifically, these map onto a global health legacy of “epidemic orientalism” (White, 2023, 10) wherein “sanitation and hygiene became signifiers, especially to Western eyes, of the cultural superiority of a salubrious West against a potential pestilential rest of the world.” Nepal's burden of modernity, it seems, is anxiously at play.

So, in the hypothetical scenario that a deadly and highly transmissible pathogen was detected in Nepal, what might we expect? In other words, if disease surveillance investments work, what do they do? Or perhaps more importantly, what do these do *either way*, even if disease surveillance may never work as intended?

## SHAMANS, PANDEMICS, AND MEDICAL MODERNITY

Unexpectedly, visiting a small homeopathic healing center on the outskirts of Kathmandu provided answers. We visited with the faint hope of meeting a “shaman” to understand what a non‐biomedical, non‐state perspective on pandemic preparedness may be. What we did not expect was that, during their lunch hour, the room would be full of an energy that felt as if we were on the brink of going out to the streets and protesting. “Allopathic medicine is the real alternative medicine!” resonated back and forth, with traditional healers vigorously expressing their frustrations at the lack of formal recognition of their practices and, more recently, the state persecution they face. In line with Peters’ (2004) ethnographic findings, they explained how *jhakris* have occupied an important role in Nepali health culture for thousands of years, echoing claims made by some health professionals who noted that the Nepali state saw healers as collaborators until recent years. Regardless, as the political tone of the conversation grew, the room insisted:

“You have to spread the word, and advocate for this kind of healing. Our healing practices have existed for thousands of years, since the origins of mankind. But the politics of allopathic medicine, which is so recent, want us to think that ours is the ‘alternative’ medicine!.”

Indeed, there has been a gradual erosion of traditional healing practices since the 1950s (Rana, 2023), resulting in tensions between these and modern medicine while subjecting the former to scrutiny of various kinds. As explained by our interlocutors, they now conduct most of their practices in secret, afraid of potential consequences should they find themselves on the wrong side of a legal system that is ambivalent towards accusations of “witchcraft,” for example (Chamling, 2021). Unsurprisingly, the public health professionals that we interviewed expressed skepticism about traditional healing practices, such as a doctor in Kathmandu who shared frustration at how hospitals sometimes receive patients bitten by rabid dogs who visited a traditional healer first, making their hospital visit too late for any potential life‐saving intervention. Among the broader Nepali public, attitudes are also shifting:

“Medical science is already advanced enough but why is our society so much a believer of *jharfuk*?^2^ Shouldn't this be illegal and direct threat to public health and public safety? What if we can make a few arrests in this regard?” (Reddit User, 2023).

Clearly, the epistemic authority of biomedical knowledge has grown, partly due to the modernization processes that Brunson (2016) describes wherein global “discourse[s] gain power at the local level (…) through [their] association with the economic supremacy of the global North.” Hence, as opposed to the health professionals we interviewed, traditional healers’ grievances were not linked to speculative notions of the future but to grounded feelings of historical injustice, frustrated by social change that has demoted them from a privileged cultural status that they see as rooted in millenary traditions. In a way, the political fervor they showed was not too different from that expressed by protestors who clamored for Nepal's “return to monarchy” in March of 2025, seen by commentators as “nostalgic”—as they called to reinstate a political system that was ousted nearly two decades ago—and stemming from enduring frustrations and a lack of belief in the future regardless of the party in power (Koirala, 2025). When the future promised by modernity gives few reasons to believe in it, nostalgia and tradition become a refuge of sorts—worthy pursuits if the alternative is becoming an outcast, as is the case for *jhakris* in contemporary Nepal.

Nevertheless, despite being accentuated by apocalyptic imaginaries of zoonotic disease emergence, such frictions between modern and traditional systems of medical knowledge are not entirely new. Rather, they are symptomatic of the broader series of civilizatory anxieties that historians of tropical medicine have described to be embedded in legacies of disease surveillance and imperial expansion. The processes at play mount back to the early 1900s, as the United States commenced an ambitious project of developing its first public health programming overseas (Anderson, 2006). The American arm of “tropical medicine” had emerged and was being tested in the Philippines, giving new meanings to the ideals of “hygiene” and “sanitation.” It was “race” which became synonymous with these. Warwick Anderson (2006, 47) recalls: “In creating a new public health in the tropics, American colonialists were commencing a ‘civilizing’ project—a ‘nation‐building’—that might, in the distant future, transform their new subjects into approximate, if not to their mind authentic, citizens.” “Sanitary inspections” of “men, manners, mind, diet, dress, and discipline” were mandated, and being a “worthy” member of modern society required “natives” to undergo sanitary reform, perceived as capable of “uplifting” an otherwise “savage” kind (Anderson, 2006, 50). The underlying ideal, Reis‐Castro (2021, 44) shows, was clear: “Putting an end to recurrent epidemics [would be] the ultimate proof that civilization [is] possible in the tropics.” Cleansing people and land from disease became the ultimate racial marker; “exotic” disease became the boundary separating worthy members of the Empire and those on whom discipline ought to be applied.

Approximately 100 years later, not much has changed. Until recently, American health investments overseas had not divested from their core ideology, motivated by the premise that “domestic action alone is insufficient to protect America's health and security” (White House, 2022, 7). Even with the United States' withdrawal from WHO and suspension of USAID funding in 2025, the project has continued. In today's multi‐polar world, “global health security” investments between 2016 and 2022 amount to over US$165 billion, of which American contributions comprise less than a third (Robertson Graeden, Kerr, Van Maele & Katz, 2024). Hence, for multiple interested parties, transnational disease surveillance networks continue to demarcate global borders and markers of belonging, operating on an epistemological platform that casts entire populations as sub‐human, “marginalized by processes that reduce them to vectors of disease” (French, 2009). Da Silva et al. (2023) ultimately argue: “In seeking and identifying certain landscapes, peoples and animals as [disease] ‘reservoirs,’ pathological associations, and racial imaginaries [are] forged between imperial, medical, and moral concerns.”

These relationships have been advanced by the introduction of One Health over the last decade, mobilizing notions of “animality” and proximity to nature in questions concerning where disease might be found. Such notions, Quijano (2000) long demonstrated, underpin a “modernity/coloniality” world system that has classified modes of being and produced hierarchies between these since the European Enlightenment. The medical arm of the expansion of modernity is not exempt, and shows of “backwardness”—as shamans and traditional healing may be interpreted by some—are in direct conflict with these modern imperatives. So, considering this, should an alarming disease be found and reported in Nepal, what may the country and its population's fate be?

Our nearest answer is found on November 25, 2021, when South African researchers publicly announced that their surveillance systems detected a new COVID‐19 variant: “Omicron.” As a result, within days, residents of several countries in southern Africa were controversially cut off from many parts of the world. The United Kingdom took the lead—quickly followed by the United States, Canada, Australia, and the European Union (EU)—in imposing a universal travel ban on passengers from seven African countries (Schermerhorn et al., 2022). The decision stirred debates, for it went against the WHO's advisory against blanket travel bans (WHO, 2021). Commentators argued: “The roots of these (…) go way back to colonial times and reflect twisted perceptions and marginalization of Africa and Africans” (Naude, 2023, 1110), while others described these bans as a “visceral reliving of the deeply painful feelings of shame, humiliation and embarrassment that the continent has been struggling to overcome since decolonization” (Naude, 2023, 1111).

These views developed as the geopolitical asymmetries in response to Omicron became clearer, when Western countries reported cases yet received no bans from other states. Travel bans, it seemed, were only applicable to African countries or ex‐colonies, leading to their later characterization as an “inappropriate punishment for strong genomic surveillance and transparent reporting” (Schermerhorn et al., 2022). Many recalled an instance when Indonesia's health minister announced that the Southeast Asian country would exercise what scholars now call “viral sovereignty” (Elbe, 2022) by refusing to share H5N1 samples with the WHO: “I saw the shadow of imperialism that had taken the most resources of my country because we had no technology to take the benefits from our resources” (Elbe, 2022, 9).

Arguably, however, for Nepal, this refusal is out of reach. Given the levels of monetary investment poured, the country is entangled in a series of transnational health dependencies that make local actors “accountable financially to USAID [and other agencies], or their board, which lies beyond the Nepalese state” (Sharma et al., 2018, 61). In a way, viral sovereignty has been renounced, and how to proceed in case of an outbreak is not an entirely local decision anymore. All parties involved “have their own interests and agendas—which are not always transparent,” Sharma, Harper, Adhikari and colleagues (2016, i3) remind us. Yet, the country's claim to belonging in modernity, mediated in this case by its healthcare system, is delicately on the line. Should the bats near the border with India and Bangladesh—an example of the “time bombs” Dr. T initially referred to—find themselves as protagonists of a large‐scale outbreak of the Nipah virus, the list of implications would be rather long. “Modernity” and its cultural politics carry weight in Nepal and in South Asia, as scholars of the region have repeatedly explored (Dave, 2012; Lukose, 2005; Nandy, 1988; Pigg, 1993).

As these discussions unfold, advances by global health actors create increasingly polar notions of risk. At the domestic level, health professionals are tasked with seeing, smelling, tasting, knowing risk, triggering cultural anxieties that are entangled with the racialized expectations of what a “civilized” and “healthy” society ought to look like. The boundaries of “the human” are re‐made in the process, extending the disparity between local health “experts” and the public they are tasked with caring for. At the international level, the millions invested by wealthy states and institutions into disease surveillance seem small given what they actually do. Seemingly, they delineate the boundaries of Empire, surveilling the world in search of microscopic evidence for “backwardness,” allegedly demarcated by the potential emergence of zoonotic diseases.

The vector, should a disease be found, could be any of poultry, pork, bats, rats, cattle, ticks, mosquitoes, or worse: a so‐called human of an “Other” race; a bio‐terrorist body waiting to complete the prophecy and put an end to Empire; one for whom “security” is the only treatment available; source of health security's epidemiological, geopolitical, cultural, and racial fears. In other words, a true zoonotic anxiety.

## CONCLUSION

In contemporary global health, pandemic preparedness and health security are largely undisputed as common sense. Anticipating the next pandemic is understood not just as a wise decision, but a political imperative. Failing to prepare when the prophecy has been foretold, possibly at the expense of millions of lives, would be hard to forgive. It is this fear that has motivated governments and organizations to invest in a global effort to avoid dealing with the messy politics of pandemic response. The way we prepare and wait, however, will not be felt only when—or if—the next pandemic arrives. There are material, cultural, and political consequences to a stance of preparedness, and these may not always be playful ghosts. Pre‐empting molecular and invisible—yet real—threats comes with equally real implications, even if hard to see. Fear, insecurity, and anxiety over the potential spread of zoonotic disease are powerful social agents, leaving a subtle trace as they pass through.

Our observations and reflections as local and foreign scholars—who are either tasked in our daily work with navigating the pressures and expectations of shifting trends in global health, have lifelong attachments to Nepal and its people, or both—attest to this. Concerns over the risks posed by zoonotic diseases are experienced differently across the country, unveiling a broad set of socio‐political orientations whenever raised. Rural and urban divides become sharper; limitations of the country's health system are inevitably discussed; the politics of military accountability are included; governing “magic” is not exempt; and, above all, Nepal's own status as modern, civilized, and worthy of participating in a cosmopolitan global society seems to be at stake. The implication is a re‐agitation of cultural dissatisfactions that sit uncomfortably in‐between Nepal's rich traditions and the country's quest toward medical modernity, alongside the widening of other local and transnational social fractures.

It would seem, then, that preparing for the “next pandemic” involves grappling with what we refer to as “zoonotic anxieties,” a complex package of fears we are tasked with coping with in relation to the imagined disaster that any animal, any time, and any place, might unleash. At its core, this frames a multispecies relational ethos that feels inevitable when the premises of contemporary global health mandates are taken seriously. But more importantly, it points to their contradictions: that, on one hand, our health is irrevocably shaped by our state of relating with the beings around us and, on the other hand, that we can shield ourselves against these. Which of these will concede first, and how might that change the broader ways we live, heal, wound, and fall ill, and ultimately relate to those around us, human and non‐human alike?

## ENDNOTES

1 This article stems from a broader multidisciplinary study conducted across Nepal to understand community‐level attitudes toward zoonotic diseases.

2
*Jharfuk* translates directly as “blowing (*fuk*) weeds (*jhar*)” or “remove (*jhar*)” and refers to the ritual procedures that shamans conduct with patients, popularly imagined to involve blowing ashes of medicinal plants on a patient to expel negative energies or spiritual afflictions.
